# Optimal sites for orthodontic anchor screw placement using panoramic images: risk of maxillary sinus perforation and contact with adjacent tooth roots during screw placement

**DOI:** 10.1186/s40510-021-00393-1

**Published:** 2021-12-08

**Authors:** Ken Miyazawa, Momoko Shibata, Masako Tabuchi, Misuzu Kawaguchi, Noriko Shimura, Shigemi Goto

**Affiliations:** Department of Orthodontics, School of Dentistry, Aichi Gakuin University, Nagoya, Japan

**Keywords:** Orthodontic anchor screws, Maxillary sinus, Safe placement

## Abstract

**Objectives:**

This study investigated the safety of orthodontic anchor screw (OAS) placement by examining the morphology and degree of depression of the maxillary sinus adjacent to the alveolar bone between the maxillary molars.

**Methods:**

We reviewed panoramic and CT imaging data of 25 patients. First, the morphology of the maxillary sinus adjacent to the alveolar bone between the maxillary molars on panoramic images was classified into three types: non-depressed sinus, funnel-like sinus depression, and sawtooth-like sinus depression. Then, the distance from the maxillary buccal bone to the maxillary sinus or to the maxillary lingual bone and the distance between the roots of the maxillary second premolar and first molar at heights of 5, 6.5, and 8 mm from the alveolar crest were measured on CT images and compared between the three sinus morphology groups.

**Results:**

The sawtooth-like depression group had significantly smaller bone thickness than the other two groups, with mean thickness of < 4 mm at any height from the alveolar crest. The funnel-like depression and non-depression groups had mean bone thickness of > 8 mm at any height from the alveolar crest.

**Conclusions:**

Sawtooth-like sinus depression had increased risk of maxillary sinus perforation, suggesting that OAS placement in this region should be avoided. In contrast, OAS placement between 6.5 and 8 mm from the alveolar crest is advisable in patients with funnel-like sinus depression and at a site > 8 mm from the alveolar crest in those with a non-depressed sinus.

## Introduction

Orthodontic anchor screws (OASs) have been introduced as an absolute source of orthodontic anchorage, enabling intrusion and distal movement of the maxillary molars, which had been considered difficult to achieve previous [1–3]. OASs are often placed in the alveolar region between the maxillary second premolar and first molar [1, 2]. However, given that the maxillary sinus floor extends between the teeth and roots adjacent to the maxillary sinus in about half of the Japanese population [4], it is important to closely examine the positional relationship between the maxillary molar alveolar bone and the maxillary sinus before OAS placement.

In patients with molar root protrusion into the maxillary sinus, there are concerns about the risk of tooth migration into the sinus during molar extraction [5] and reduced thickness of the alveolar bone for dental implant placement after extraction [6]. From an orthodontic perspective, the risk of tipping and root resorption during distal movement of the maxillary molars has been reported [7, 8].

Given these concerns, the positional relationship between the maxillary molars and the maxillary sinus has been extensively examined [9–11]. However, these reports focused on this positional relationship and did not morphologically characterize the maxillary sinus floor adjacent to the alveolar bone between the maxillary molars. Because an OAS is often placed in the alveolar region between the maxillary second premolar and first molar, we considered it important to evaluate the positional relationship of the alveolar bone between the maxillary molars and the maxillary sinus in order to ensure safe OAS placement.

In this study, we propose a new classification for the morphology of the maxillary sinus in the maxillary molar alveolar region on panoramic images and then closely examined the maxillary sinus on CT images to evaluate the risk of maxillary sinus perforation and contact with adjacent tooth roots during OAS placement.

## Methods and materials

### Patients

We analyzed panoramic and CT image data of 25 patients (50 sites, including left and right) who visited our hospital between May 2008 and October 2011 and were diagnosed as requiring OAS placement for orthodontic treatment. The patients comprised 24 women and 1 man, with mean age of 22.3 (range 14–39) years.

The inclusion criteria were as follows: no congenital diseases, such as cheilognathopalatoschisis; no obvious deformity of the maxilla, including asymmetry; completed jaw growth, full dentition with no missing teeth, and no severe periodontal disease; and availability of CT images of sufficient quality to allow for precise measurement.

This study was approved by the ethics committee of our university (approval no. 243) and was conducted in accordance with the Declaration of Helsinki. All patients provided written informed consent to participate.

### Panoramic and CT imaging

Panoramic X-ray images were taken with a Veraviewepocs X550 panoramic unit (Morita Corporation, Kyoto, Japan) using an imaging plate at 75 kV and 8 mA, and the digital panoramic images were output to film (Drypro Model 793, Konica Minolta, Tokyo, Japan). CT scans were taken with an Asteion system (Toshiba Medical Systems Corporation, Tokyo, Japan). The patient was positioned in the supine position with the mouth closed and the occlusal plane perpendicular to the floor. Tube voltage was set to 120 kV, tube current to 150 mA, field of view to 20 cm, and slice thickness to 0.5 mm. The imaging field extended from the inferior orbital margin to the occlusal plane. The obtained CT data were stored in DICOM format on a portable hard disk.

### Analysis of imaging data

**Fig. 1:**
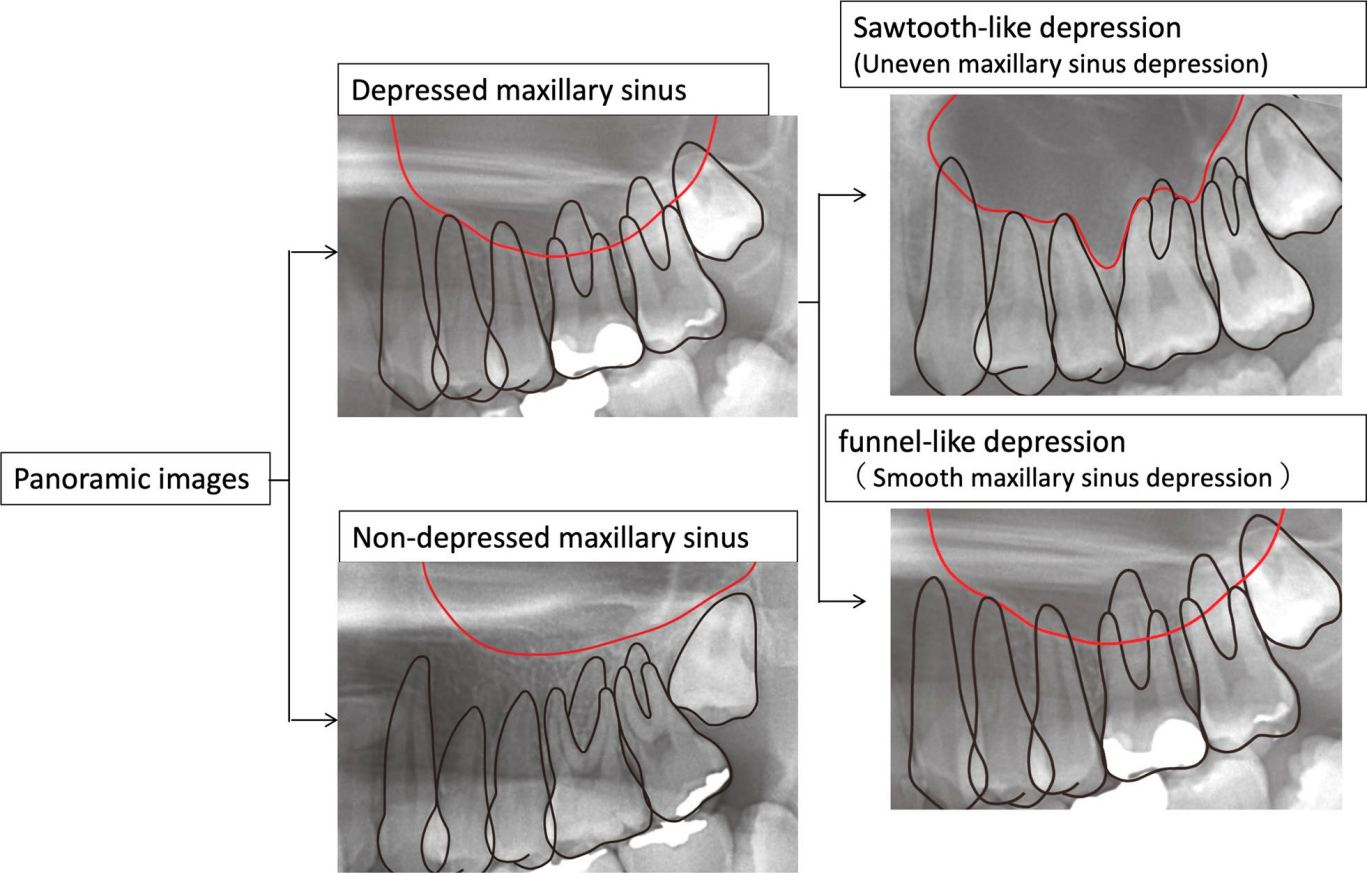
Classification of maxillary sinus morphology on panoramic images. On panoramic images, the maxillary sinus morphology was classified into two types: one in which the sinus floor was depressed such that it was adjacent to the alveolar bone between the maxillary molars (depressed sinus type) and the other in which it was not depressed (non-depressed sinus type). The depressed sinus type was further classified according to the sinus floor morphology into two types: the sawtooth-like depression type and the funnel-like depression type

The acquired panoramic cephalograms were used to classify the maxillary sinus morphology into two types: one in which the sinus floor was depressed such that it was adjacent to the alveolar bone between the maxillary molars (depressed sinus) and the other in which it was not depressed (non-depressed sinus). The depressed type was further classified according to the shape of the maxillary sinus floor into the funnel-like and sawtooth-like depression types (Fig. 1).

**Fig. 2:**
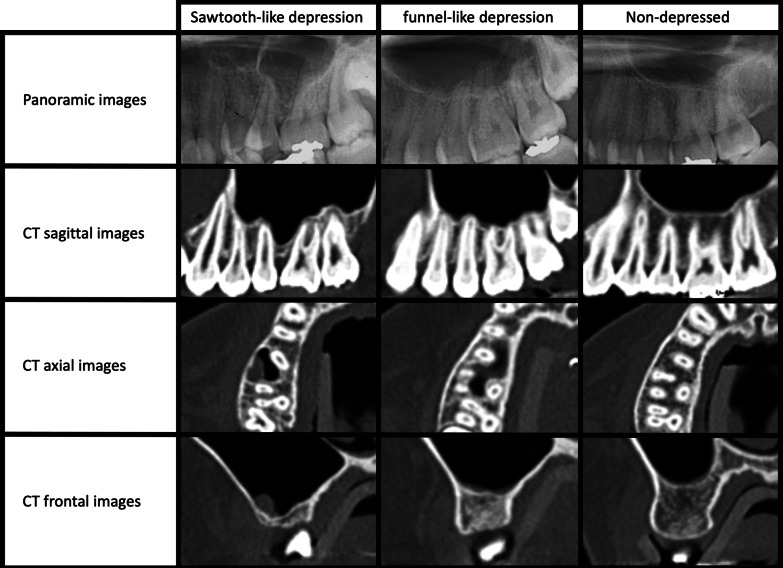
Representative panoramic images and their corresponding CT images

We then used three-dimensional (3D) image processing software (OsiriX, Geneva, Switzerland) and a personal computer (Macbook Pro, Apple Computer, Cupertino, CA) to create 3D images from the obtained CT data. Figure 2 shows representative panoramic images and their corresponding CT images.

**Fig. 3:**
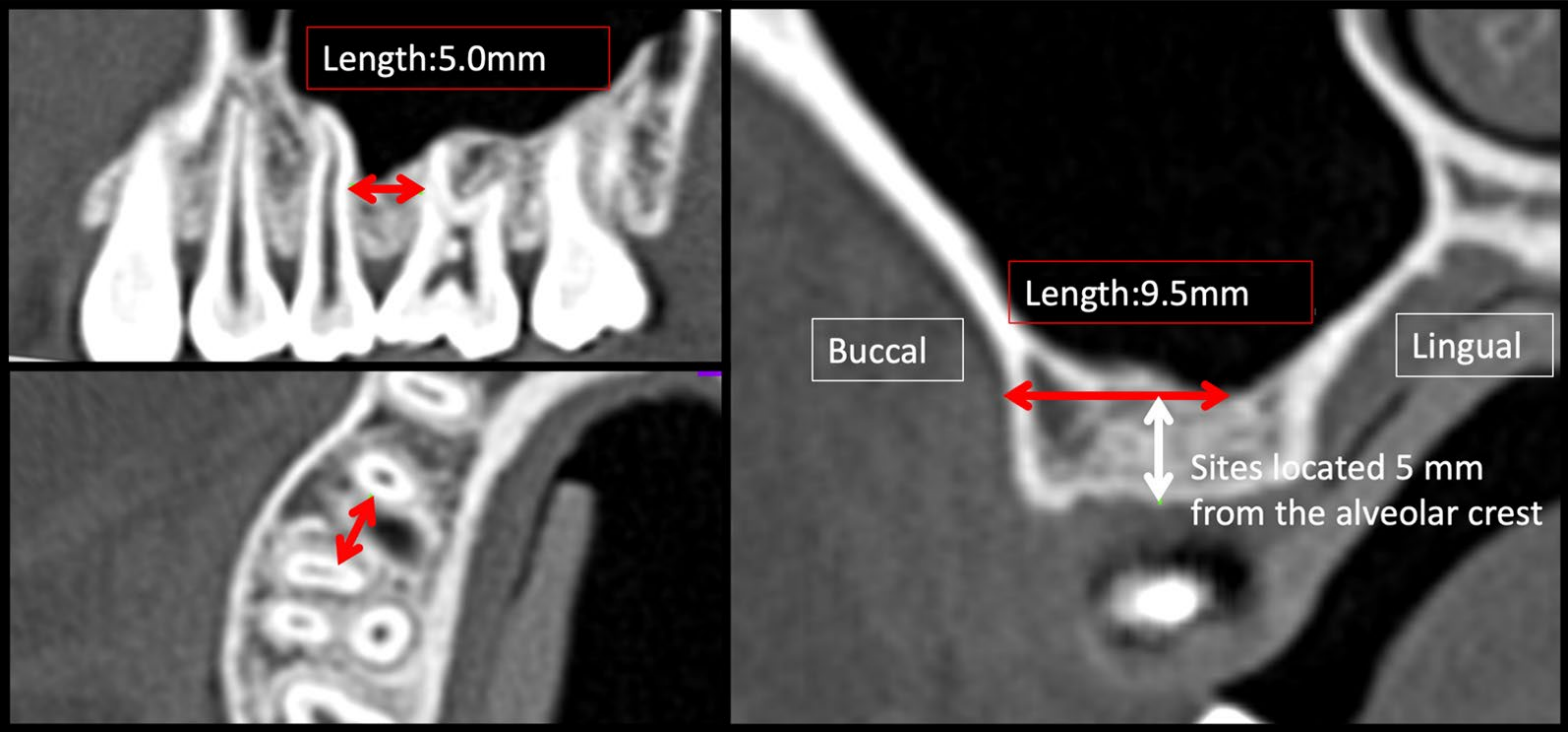
Measurements on CT images. Maxillary buccal alveolar bone thickness and distance between the roots of the maxillary second premolar and first molar at 5, 6.5, and 8 mm from the alveolar crest were measured on CT images

On CT cross section parallel to the occlusal plane, the distance from the maxillary buccal bone to the maxillary sinus or to the maxillary lingual bone and the distance between the roots of the maxillary second premolar and first molar at heights of 5, 6.5, and 8 mm from the alveolar were measured (Fig. 3) and compared between the three sinus morphology groups (non-depression, funnel-like depression, and sawtooth-like depression).

### Sample size calculation

We planned to study a continuous response variable in independent control and experimental subjects with control per experimental subject. In a previous study, the response within each subject group was normally distributed with standard deviation 1 mm. Assuming the true difference between the experimental and control means is 2 mm, we would need 5 experimental subjects and 5 control subjects to be able to reject the null hypothesis that the population means of the experimental and control groups are equal with probability (power) 0.8. The probability of Type I error associated with this test of the null hypothesis is 0.05.

### Statistical analysis

Statistical analyses were performed using JMP 12.0.1 (SAS Institute. Cary, NC).

Data were first subjected to the Shapiro–Wilk test to assess normality, and then to the Tukey–Kramer test if the data were normally distributed or the Steel–Dwass test if not. A p-value less than 0.05 was considered statistically significant.

## Results

Table 1 shows the mean and standard deviation of buccolingual bone thickness and inter-radicular distance at 5, 6.5, and 8 mm from the alveolar crest according to sinus morphology type.

**Table 1:** Buccolingual bone thickness and inter-radicular distance at various heights from the alveolar crest between the maxillary second premolar and first molar

	N	Height from alveolar crest		
		5 mm	6.5 mm	8 mm
Bone thickness (mm)				
Sawtooth-like depression	6	4.0 ± 2.6	1.9 ± 1.1	1.6 ± 0.5
Funnel-like depression	25	11.3 ± 1.9	10.8 ± 3.2	8.8 ± 4.4
Non-depression	19	12.2 ± 1.4	12.2 ± 1.4	12.5 ± 1.3
Inter-radicular distance (mm)				
Sawtooth-like depression	6	4.2 ± 0.6	4.5 ± 0.7	5.3 ± 0.9
Funnel-like depression	25	2.7 ± 0.6	3.0 ± 0.7	3.6 ± 0.9
Non-depression	19	2.9 ± 0.8	3.1 ± 0.8	3.6 ± 0.9

Values are shown as the mean ± standard deviation

**Fig. 4:**
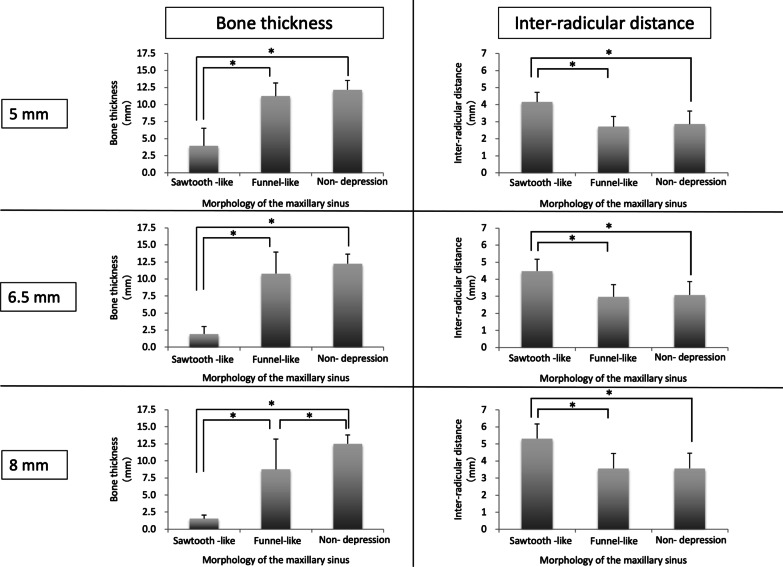
Comparison of buccolingual bone thickness and inter-radicular distance between the three sinus morphology types at 5, 6.5, and 8 mm from the alveolar crest between the maxillary second premolar and first molar

**Fig. 5:**
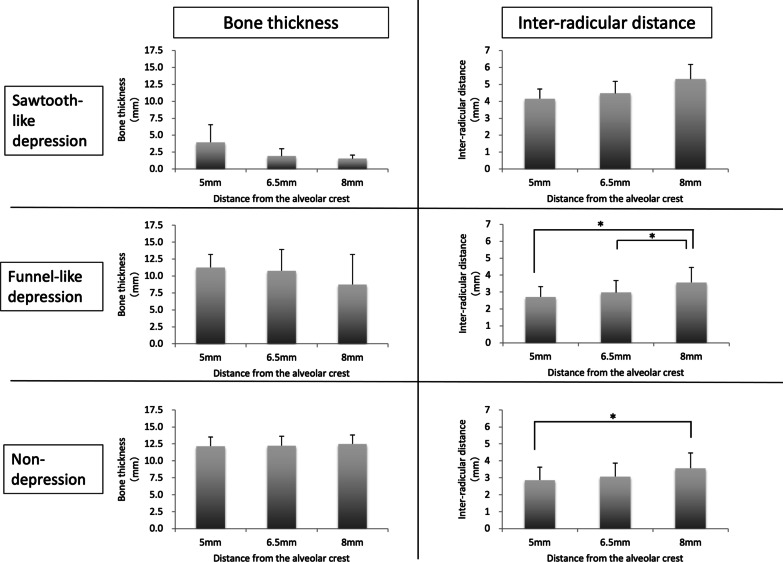
Comparison of buccolingual bone thickness and inter-radicular distance by distance from the alveolar crest in each sinus morphology type

Figure 4 shows a comparison of buccolingual bone thickness and inter-radicular distance between the three sinus morphology types at each site, while Fig. 5 shows a comparison of buccolingual bone thickness and inter-radicular distance by distance from the alveolar crest in each sinus morphology type. The sawtooth-like depression group had a significantly smaller bone thickness from the maxillary buccal bone to the maxillary sinus than the other two groups, with mean thickness of < 4 mm at any height from the alveolar crest. On the other hand, the inter-radicular distance at the maxillary second premolar/first molar was > 4 mm at any height. The funnel-like depression and non-depression groups had a significantly smaller inter-radicular distance compared with the sawtooth-like depression group. In the funnel-like depression group, mean buccolingual bone thickness was < 8 mm at any height and mean inter-radicular distance was 2.7, 3.0, and 3.6 mm at heights of 5, 6.5, and 8 mm from the alveolar crest, respectively. The corresponding distance in the non-depression group was 2.9, 3.1, and 3.6 mm, respectively.

## Discussion

Orthodontic treatment with the use of OAS as an absolute source of anchorage has become increasingly popular [1–3, 12, 13]. The most commonly reported sites for OAS placement in the maxilla are the buccal molar inter-radicular region [1, 2] and the median palate [3, 12, 13]. OAS placement in the buccal molar inter-radicular region is usually done with one screw each on the right and left sides. However, this procedure is associated with a risk of root damage or perforation into the maxillary sinus [14, 15]. Moreover, the deep part of the buccal alveolar region is covered by the movable mucosa and susceptible to inflammation.

Wey et al. [16] stated that the safest site for OAS placement in Mongoloids is 5–8 mm from the cement-enamel junction between the maxillary second premolar and first molar, while Poggio et al. [17] stated that an inter-radicular distance at the maxillary second premolar/first molar of at least 3.1 mm allows for the safe placement of OAS of 1.2–1.3 mm in diameter. Given that most OASs currently in use are 5–8.5 mm in length and 1.2–2.0 mm in diameter [1–3, 12, 13, 17], an inter-radicular distance of at least 3.1 mm and a bone thickness from the buccal bone to the maxillary sinus of at least 8 mm must be secured for safe OAS placement between the maxillary second premolar and first molar. However, the outcome of OAS placement in this region may depend on how the maxillary sinus is depressed, which is an important issue to keep in mind also in terms of the risk of maxillary sinus perforation and infection.

In this study, in order to determine the optimal sites for safe OAS placement, we first classified the morphology of the maxillary sinus in the maxillary molar alveolar region on panoramic radiographs into three types: sawtooth-like sinus depression, funnel-like sinus depression, and non-depressed sinus. We then precisely measured the inter-radicular distance at the maxillary second premolar/first molar and the distance from the maxillary buccal bone to the maxillary sinus on CT images.

The results showed that the sawtooth-like depression group had a significantly smaller bone thickness from the maxillary buccal bone to the maxillary sinus than the funnel-like depression and non-depression groups, with mean thickness of < 4 mm at any height from the alveolar crest. This suggests that miniscrew placement in the alveolar region between the maxillary second premolar and first molar should be avoided in patients with sawtooth-like sinus depression due to the extremely high risk of maxillary sinus perforation. Alternative strategies are needed in these cases, such as changing the placement site to the maxillary palate.

In the funnel-like depression group, mean buccolingual bone thickness was < 10 mm at a distance of 5 and 6.5 mm from the alveolar crest and slightly more than 8 mm at a distance of 8 mm, suggesting that OAS placement at a site closer to the root apex than the 8-mm site is associated with a risk of maxillary sinus perforation.

On the other hand, mean inter-radicular distance at the maxillary second premolar/first molar was 2.7, 3.0, and 3.6 mm at heights of 5, 6.5, and 8 mm from the alveolar crest, respectively. It is therefore considered safe to place an OAS between 6.5 mm and 8 mm from the alveolar crest in patients with funnel-like sinus depression. OAS placement at a site closer to the alveolar crest than the 6.5-mm site may increase the risk of the screw contact with the adjacent tooth roots, while placement at a site closer to the root apex than the 8-mm site is associated with a risk of maxillary sinus perforation.

In the non-depression group, there was sufficient buccolingual bone thickness for OAS placement at any site, with no significant difference in bone thickness between sites. However, an average inter-radicular distance at the maxillary second premolar/first molar of at least 3.1 mm, which is required for safe OAS placement as described by Poggio et al. [17], was secured only at 8 mm from the alveolar crest. Thus, in patients with non-depressed sinus, the inter-radicular distance at the maxillary second premolar/first molar is smaller than that in those with sawtooth-like sinus depression, and it is considered safe to place an OAS as far as possible from the alveolar crest (at least 8 mm), in other words, closer to the root apex.

In clinical practice, inserting an OAS with a certain amount of angulation will reduce the risk of perforation because the distance to the maxillary sinus will be longer than that achieved in this study. However, in this study, we assumed a situation where there was high risk of maxillary sinus perforation by deliberately setting the insertion angle horizontal, and we investigated the safety of OAS placement in this situation. Bower [18] and Loe et al. [19] reported that the width of the attached gingiva in the maxillary molar region is approximately 4 to 5 mm. This suggests that it is safer to insert an OAS with angulation from the attached gingiva into a deeper space.

Panoramic radiography is the most common radiographic modality used in general dental practices to examine the maxillary sinus. However, it is often difficult to examine the maxillary sinus and surrounding structures because structures such as the hard palate and inferior nasal concha overlap with the maxillary sinus [20] and posterior structures far from the tomographic layer are not clearly depicted. Ohashi et al. reported that for dentists with < 2 years of clinical experience, the use of panoramic radiographs alone for diagnosing maxillary sinusitis resulted in a correct diagnosis rate of only 66.0% and sensitivity of 63.4%, giving lower diagnostic performance compared with the use of computer-assisted diagnosis system [21].

In terms of the positional relationship between the maxillary sinus and the maxillary molar roots, Sharan et al. reported that only 39% of the patients with root protrusion into the maxillary sinus on panoramic images actually had the protrusion on CT images. At the same time, patients with no maxillary molar root protrusion on panoramic images also had no maxillary molar root protrusion on CT images, and thus, the authors concluded that panoramic radiographs are sufficient to understand the actual relationship between the maxillary molar roots and the maxillary sinus in these patients [9]. However, these findings apply to only the positional relationship between the maxillary molar roots and the maxillary sinus, and caution should be exercised when placing an OAS in the alveolar bone between the maxillary molars.

Our data show that the inter-radicular distance at the maxillary second premolar/first molar tended to be wider in the sawtooth-like depression group than those in the other two groups, but the bone thickness from the buccal bone to the maxillary sinus was extremely small, making it almost impossible to place an OAS in the maxillary buccal alveolar bone. Therefore, in patients with sawtooth-like sinus depression on panoramic images, OAS placement in the maxillary buccal alveolar bone should be avoided and other strategies should be considered, such as placing the OAS in the maxillary median palate, where there is sufficient bone thickness and no risk of root damage. In contrast, as mentioned above, OAS placement is considered safe between 6.5 and 8 mm from the alveolar crest in patients with panoramic radiographic evidence of funnel-like sinus depression and at a site > 8 mm from the alveolar crest in those with non-depressed sinus. In this respect, panoramic images are useful as a screening tool for the safe placement of OASs. However, the best way to ensure safe OAS placement would be to obtain panoramic, dental, and 3D radiographs (CT or CBCT) prior to OAS placement. (CT was used in this study.) However, we conducted this study to test the hypothesis that the positional relationship between the site of OAS placement and the maxillary sinus can be evaluated on panoramic images to identify patients at low risk of maxillary sinus perforation in whom OAS placement can be done without acquiring 3D images, which would help reduce radiation exposure. We also believe that 3D images should be taken if perforation into the maxillary sinus or contact with the adjacent tooth root is suspected from panoramic X-ray findings. When large-diameter OASs are to be used or when patients have a small inter-radicular distance on panoramic images, careful consideration is required when placing the OAS, such as the use of a stent and CT images [22]. Further studies with a larger sample size are necessary to evaluate the possible associations revealed here.

## Conclusion

Sawtooth-like maxillary sinus depression was associated with significantly smaller buccolingual bone thickness at any height from the alveolar crest and thus an increased risk of maxillary sinus perforation compared with the other two types, suggesting that OAS placement in this region should be avoided. In contrast, the funnel-like depression and non-depression types had a significantly smaller inter-radicular distance compared with the sawtooth-like depression type, suggesting that the risk of the contact with adjacent roots increases as the site of OAS placement becomes closer to the alveolar crest. Therefore, OAS placement between 6.5 and 8 mm from the alveolar crest is advisable in patients with funnel-like sinus depression and at a site > 8 mm from the alveolar crest in those with non-depressed sinus.

## Data Availability

The data underlying this article will be shared on reasonable request to the corresponding author.

## References

[1] Chung KR, Choo H, Kim SH, Ngan P (2010). Timely relocation of mini-implants for uninterrupted full-arch distalization. Am J Orthod Dentofac Orthop.

[2] Park HS, Kwon TG (2004). Sliding mechanics with microscrew implant anchorage. Angle Orthod.

[3] Shibata M, Miyazawa K, Tabuchi M, Kawaguchi M, Shimura N, Takeguchi A, Goto S (2016). Maxillary molar distalization and intrusion achieved with an orthodontic anchoring screw (OAS) placed in the mid-palatal area and a modified palatal bar in a patient with maxillary prognathism. Aichi-Gakuin J Dental Sci.

[4] Hauman CH, Chandler NP, Tong DC (2002). Endodontic implications of the maxillary sinus: a review. Int Endod J.

[5] Harrison DF (1961). Oro-antral fistula. Br J Clin Pract.

[6] Wehrbein H, Diedrich P (1992). Progressive pneumatization of the basal maxillary sinus after extraction and space closure [in German]. Fortschr Kieferorthop.

[7] Daimaruya T, Takahashi I, Nagasaka H, Umemori M, Sugawara J, Mitani H (2003). Effects of maxillary molar intrusion on the nasal floor and tooth root using the skeletal anchorage system in dogs. Angle Orthod.

[8] Wehrbein H, Bauer W, Wessing G, Diedrich P (1990). The effect of the maxillary sinus floor on orthodontic tooth movement [in German]. Fortschr Kieferorthop.

[9] Sharan A, Madjar D (2006). Correlation between maxillary sinus floor topography and related root position of posterior teeth using panoramic and cross-sectional computed tomography imaging. Oral Surg Oral Med Oral Pathol Oral Radiol Endod.

[10] Kwak HH, Park HD, Yoon HR, Kang MK, Koh KS, Kim HJ (2004). Topographic anatomy of the inferior wall of the maxillary sinus in Koreans. Int J Oral Maxillofac Surg.

[11] Kilic C, Kamburoglu K, Yuksel SP, Ozen T (2010). An assessment of the relationship between the maxillary sinus floor and the maxillary posterior teeth root tips using dental cone-beam computerized tomography. Eur J Dent.

[12] Lee J, Miyazawa K, Tabuchi M, Kawaguchi M, Shibata M, Goto S (2013). Midpalatal miniscrews and high-pull headgear for anteroposterior and vertical anchorage control: cephalometric comparisons of treatment changes. Am J Orthod Dentofac Orthop.

[13] Lee J, Miyazawa K, Tabuchi M, Sato T, Kawaguchi M, Goto S (2014). Effectiveness of en-masse retraction using midpalatal miniscrews and a modified transpalatal arch: treatment duration and dentoskeletal changes. Korean J Orthodont.

[14] Asscherickx K, Vannet BV, Wehrbein H, Sabzevar MM (2005). Root repair after injury from mini-screw. Clin Oral Implant Res.

[15] Motoyoshi M, Sanuki-Suzuki R, Uchida Y, Saiki A, Shimizu N (2015). Maxillary sinus perforation by orthodontic anchor screws. J Oral Sci.

[16] Wey MC, Shim CN, Lee MY, Jamaluddin M, Ngeow WC (2012). The safety zone for mini-implant maxillary anchorage in Mongoloids. Aust Orthod J.

[17] Poggio PM, Incorvati C, Velo S, Carano A (2006). “Safe Zones”: a guide for miniscrew positioning in the maxillary and mandibular arch. Angle Orthod.

[18] Bowers G (1963). A study of the width of attached gingiva. J Periodontol.

[19] Ainamo J, Loe H (1966). Anatomical charactaristics ofgingiva. A clinical and microscopic study of the free and attached gingiva. J Periodontol.

[20] Damante JH, Filho LI, Silva MA (1998). Radiographic image of the hard palate and nasal fossa floor in panoramic radiography. Oral Surg Oral Med Oral Pathol Oral Radiol Endod.

[21] Ohashi Y, Ariji Y, Katsumata A, Fujita H, Nakayama M, Fukuda M, Nozawa M, Ariji E (2016). Utilization of computer-aided detection system in diagnosing unilateral maxillary sinusitis on panoramic radiographs. Dentomaxillofac Radiol.

[22] Miyazawa K, Kawaguchi M, Tabuchi M, Goto S (2010). Accurate pre-surgical determination for self-drilling miniscrew implant placement using surgical guides and cone-beam computed tomography. Eur J Orthod.

